# Active Human and Murine Tumor Necrosis Factor α Cytokines Produced from Silkworm Baculovirus Expression System

**DOI:** 10.3390/insects12060517

**Published:** 2021-06-02

**Authors:** Takeru Ebihara, Jian Xu, Yoshino Tonooka, Takumi Nagasato, Kohei Kakino, Akitsu Masuda, Kosuke Minamihata, Noriho Kamiya, Hirokazu Nakatake, Yuuka Chieda, Hiroaki Mon, Tsuguru Fujii, Takahiro Kusakabe, Jae Man Lee

**Affiliations:** 1 Laboratory of Insect Genome Science, Kyushu University Graduate School of Bioresource and Bioenvironmental Sciences, Motooka 744, Nishi-ku, Fukuoka 819-0395, Japan; ebihara0915@agr.kyushu-u.ac.jp (T.E.); y.tonooka@agr.kyushu-u.ac.jp (Y.T.); nagasato@agr.kyushu-u.ac.jp (T.N.); k.kakino@agr.kyushu-u.ac.jp (K.K.); a.masuda@agr.kyushu-u.ac.jp (A.M.); mhiro@agr.kyushu-u.ac.jp (H.M.); kusakabe@agr.kyushu-u.ac.jp (T.K.); 2 Laboratory of Biology and Information Science, Biomedical Synthetic Biology Research Center, School of Life Sciences, East China Normal University, Shanghai 200062, China; 3 Department of Applied Chemistry, Graduate School of Engineering, Kyushu University, Motooka 744, Nishi-ku, Fukuoka 819-0395, Japan; minamihata.kosuke.224@m.kyushu-u.ac.jp (K.M.); kamiya.noriho.367@m.kyushu-u.ac.jp (N.K.); 4 Division of Biotechnology, Center for Future Chemistry, Kyushu University, Motooka 744, Nishi-ku, Fukuoka 819-0395, Japan; 5 KAICO Ltd., 4-1, Kyudaishinmachi, Nishi-ku, Fukuoka 819-0388, Japan; nakatake@kaicoltd.jp (H.N.); iiyama@kaicoltd.jp (Y.C.); 6 Laboratory of Creative Science for Insect Industries, Kyushu University Graduate School of Bioresource and Bioenvironmental Sciences, Motooka 744, Nishi-ku, Fukuoka 819-0395, Japan; fujii.tsuguru.233@m.kyushu-u.ac.jp

**Keywords:** TNFα, protein purification, baculovirus, silkworm expression system

## Abstract

**Simple Summary:**

Baculovirus expression vector system (BEVS) is widely employed to produce eukaryotic recombinant proteins with desired post-translational modifications. The tumor necrosis factor α (TNFα) is a promising reagent in treating autoimmunity and cancer diseases. In the current study, we designed to express and purify human and murine TNFα proteins in a silkworm larva-based baculovirus expression vector system (silkworm-BEVS). The results demonstrated that the desirable productivity of proteins with similar biological activity was experimentally confirmed. It was revealed that the C-terminal fusion tags negatively impacted their biological activity, as confirmed in the cytotoxicity assay. Taken together, silkworm-BEVS is an alternative platform for supplying high-quality TNFα products for various purposes.

**Abstract:**

The tumor necrosis factor α (TNFα) has been employed as a promising reagent in treating autoimmunity and cancer diseases. To meet the substantial requirement of TNFα proteins, we report in this study that mature types of recombinant human and murine TNFα proteins are successfully expressed in the baculovirus expression system using silkworm larvae as hosts. The biological activities of purified products were verified in culture murine L929 cells, showing better performance over a commercial *Escherichia coli*-derived murine TNFα. By comparing the activity of purified TNFα with or without the tag removal, it is also concluded that the overall activity of purified TNFα cytokines could be further improved by the complete removal of C-terminal fusion tags. Collectively, our current attempt demonstrates an alternative platform for supplying high-quality TNFα products with excellent activities for further pharmaceutical and clinical trials.

## 1. Introduction

The tumor necrosis factor α (TNFα) is a pleiotropic cytokine produced from activated macrophages and lymphocytes that regulates both pro-inflammatory responses and cellular communications [1,2]. The aberrant expression of TNFα is usually correlated with some autoimmune diseases, such as rheumatoid arthritis, Chron’s disease, and atherosclerosis [2,3,4]. To date, the TNFα gene or protein has already been described in mammals, fish, amphibians, and recently, also in the avian genomes [3,5]. The TNFα members in mammals such as a human (hTNFα) and murine (mTNFα) are type II transmembrane proteins presented in either a membrane-bound (~26 kDa) or a free soluble (~17 kDa) homotrimer after proteolytic cleavage by a TNFα-converting enzyme (TACE) [4,6,7]. The most attractive function discovered of soluble TNFα protein is the cytotoxic activity against specific tumor cells in vitro and in vivo, mainly through binding to and activating one of its two distinct membrane receptors, TNFR1 [2,3]. The killing effects of the TNFR1 signaling pathway endows the mature type of TNFα a potential drug for the treatment of cancer in the clinical trial market. Because of its high demand, the productions of recombinant human and murine TNFα proteins with desirable functions have been investigated in various heterologous protein expression systems, including *Escherichia coli*, *Streptomyces lividans*, *Spodoptera frugiperda* Sf9 cells, or mouse embryonic 3T3 fibroblasts [8,9,10,11,12]. To achieve better productivity of TNFα, there have been constant efforts in optimizations in the genetic gene codon, expression promoters, culture, and induction conditions of the *E. coli* expression system targeting more soluble protein products over inclusion body in most cases [8,9,13,14,15]. The eukaryotic protein expression systems such as mammalian cell expression systems and baculovirus expression vector system (BEVS) have advantages in achieving soluble targets without frustrating condition optimizations, which seems necessary in the *E. coli* system, although shortages also exist regarding the relatively higher cost due to cell culture and laboratory maintenance [16,17,18].

High productivity of protein with reasonable post-translational modifications and great flexibility for large protein complexes are attractive hallmarks of BEVS using either lepidopteran cells or insects such as the domestic silkworm, *Bombyx mori*, one of the critical economic insects in many Asian countries [18,19,20,21]. It is the primary source of the silk industry and is also recognized as a model organism for fundamental research. Remarkably, additional explorations of suitable combinations of a nucleopolyhedrovirus (NPV) and larva or pupa host strains as a proficient silkworm-BEVS bioreactor have been extensively performed recently [18,22,23,24]. Low cost, high yields, and few ethical issues are recognizable as the main merits of silkworm-BEVS. Thus, we designed to express and purify both the human and murine TNFα proteins in silkworm-BEVS. After achieving a fair amount of the purified proteins, we investigated the productivity and biological activity in culture murine L929 cells. Moreover, we also removed the C-terminal fusion tags and validated that those tags negatively impacted their biological activity. Taken together, our current attempt demonstrates an alternative platform for supplying high-quality TNFα products for further cytokine-related academic research and pharmaceutical clinical trials.

## 2. Materials and Methods

### 2.1. Cells and Silkworms

The silkworm BmN (*Bombyx mori*-derived cells, Funakoshi Inc., Tokyo, Japan) cell line was stably cultured in an IPL-41 medium (Sigma, St. Louis, MO, USA) supplemented with 10% fetal bovine serum (FBS, Gibco, Grand Island, NY, USA) at 27 °C. The silkworm k45 silkworm strain was provided by the Institute of Genetic Resources, Kyushu University Graduate School (Japan National BioResource Project). The insect larvae were reared on fresh mulberry leaves under well-controlled environmental conditions at 25–27 °C.

### 2.2. Construction of Recombinant Baculoviruses

**Figure 1:**
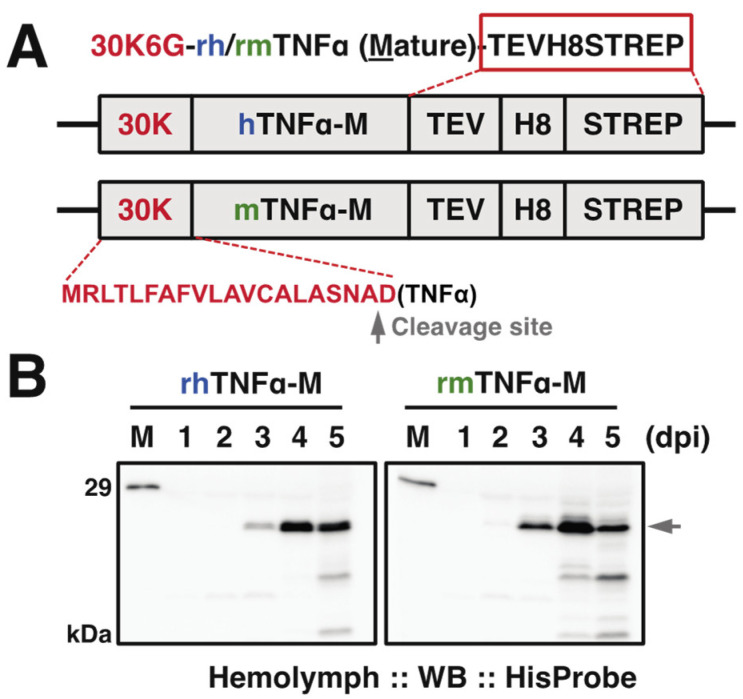
(**A**) Expression cassette of the recombinant baculovirus for hTNFα and mTNFα. pDEST8-hTNFα or -mTNFα was employed to generate the recombinant baculovirus (strain Q4), respectively. 30K6G (30K): a signal peptide from silkworm endogenous 30K protein 6G for the sufficient secretion of TNFα. A gray arrowhead indicated the predicted cleavage site for 30K signal peptide by SignalP 5.0 (http://www.cbs.dtu.dk/services/SignalP/(accessed on 29 May 2021)). hTNFα: mature type of human TNFα; mature type of mTNFα: murine TNFα, TEV: tobacco etch virus protease cleavage site; H8: 8x Histidine tag; STREP: Strep-tag. C-terminal tags were marked in the red rectangle. (**B**) Time course of expressed TNFα in hemolymph from baculovirus-infected silkworm larvae at indicated day post-infection (dpi). Western blot was done using anti-HisProbe, and the arrowhead indicated the expressed TNFα in full length. M: molecular mass markers.

Total RNAs from the kidney of ICR mice (Charles River, Yokohama, Japan) and human (Stratagene, La Jolla, San Diego, CA, USA) were used for first-strand cDNA synthesis with SuperScript II RNase H-reverse transcriptase (Invitrogen, Carlsbad, CA) and oligo-(dT) primer. To construct gateway-based entry clones, the open-reading frame (ORF) of Mus musculus TNFα (mTNFα, amino acids 80-235 aa: GenBank accession number, NM_013693.3) and human TNFα (hTNFα, amino acids 77-233aa: GenBank accession number, NM_000594.4) was amplified by polymerase chain reaction (PCR) with KOD-Plus-Neo DNA polymerase (TOYOBO, Tokyo, Japan), respectively. The primers used for the PCR reactions were mTNFα-M-5 (5′-CTCAGATCATCTTCTCAAAATTCGAGTGAC-3′), mTNFα-M-3-XhoI (5′-ggggCTCGAGAGAGCAATGACTCCAAAGTAG-3′) and hTNFα-M-5 (5′-GTCAGATCATCTTCTCGAACCCCGAGTG-3′), hTNFα-M-3-XhoI (5′-ggggCTCGAGAGGGCAATGATCCCAAAGTAG-3′), respectively. The *Xho*I-digested amplicon was inserted into the modified pENTR11 (*Xho*I digested pENTRL21-30K-TEVH8STREP amplicon; our laboratory stocks) vector by Ligation High (TOYOBO, Tokyo, Japan). As described previously, a lobster L21 sequence was employed to enhance translations in BEVS [25], and C-terminal His8-STREP tags were used to facilitate the purification of proteins of interest (POIs) [26,27]. As illustrated in Figure 1A, the resulting constructs pENTR-L21-30K-rh/rmTNFα (Mature)-TEVH8STREP were then incorporated into the pDEST8 vector (Invitrogen, Carlsbad, CA) by the Gateway LR reaction to generate baculovirus transfer plasmid following the manufacturer’s protocol. Recombinant h/mTNFα baculoviruses were created using a bacmid DNA of *B. mori* nucleopolyhedrovirus (BmNPV) Qd04 strain [24] described previously by Ono et al. [28]. The bacmid DNA was then transfected into the BmN cells by a FuGENE HD transfection reagent (Promega, Madison, WI) to generate the recombinant virus particles (BmNPV/polh-30K-rh/rmTNFα-TEVH8STREP). The culture supernatant was harvested as the P1 virus on the 4th day after cell transfection. The high-titer virus (P3) stock was prepared after a serial infection of baculovirus in cultured cells. All viruses for silkworm infections were kept at 4 °C in the dark until use.

### 2.3. Expression and Purification of rhTNFα and rmTNFα in Silkworm Larvae

To confirm the expression of rh/rmTNFα in the silkworm-BEVS, the recombinant viruses (~1 × 10^5^ plaque-forming unit per larvae) were injected into the 5th instar silkworm larvae (day 3) of the k45 silkworm strain. On the fourth day after injection, the sera were collected into a tube containing 20 mM 1-phenyl-2-thiourea, which were then cleared by centrifugation at 8500 rpm for 30 min at 4 °C. The supernatant of serum was subjected to a two-step purification procedure for the rhTNFα and rmTNFα protein, respectively. The detailed sequential His- and Strep-tag chromatography processes were adopted, as developed previously [29]. Briefly, the collected sera samples were diluted in a binding buffer (20 mM Tris–HCl pH 7.4, 0.5 M NaCl, and 20 mM 1-phenyl-2-thiourea) and centrifuged followed by clarification through a 0.45 μm filter (Millipore, Boston, MA, USA). The protein sample was then loaded onto a 5 mL HisTrap excel column (GE Healthcare Bioscience, Piscataway, NJ, USA). The His-tagged proteins were eluted with the elution buffer (20 mM Tris-HCl pH7.5 and 0.5M NaCl) containing 100 mM and 500 mM imidazole. Subsequently, the fractions containing TNFα proteins were collected and concentrated by ultrafiltration using Amicon Ultra-15 3 K filters (Millipore, Boston, MA, USA). The concentrated proteins were then diluted in a PBS buffer and further loaded onto a 5 mL Strep-Tactin HP column (IBA GmbH, Göttingen, Germany) for a second purification. The rTNFα proteins were eluted with a PBS buffer containing 2.5 mM desthiobiotin. Elution fractions were also concentrated using Amicon 3 K filters (Millipore, Boston, MA, USA). The final protein yield of rh/rmTNFα was determined by YabGelImage software (https://sites.google.com/site/yabgel/(accessed on 25 July 2020) using bovine serum albumin (BSA) as a standard. All protein samples were separated on 12% SDS-PAGE and visualized by Coomassie Brilliant Blue (CBB) R-250 staining.

### 2.4. Removal of Terminal Fusion Tags from Purified rh/mTNFα Proteins

To investigate whether or not C-terminal tags have a negative impact on TNFα protein functions, TEV proteinase (His-tag-fused, a laboratory stock produced from silkworm-BEVS) was employed to remove the TEV-H8-STREP fusion tags at C-terminus. Briefly, the purified recombinant hTNFα or mTNFα protein (~2 mg) was mixed with the TEV proteinase (~2 mg) in a TEV cleavage buffer (50 mM Tris-HCl pH 8.0, 0.5 mM EDTA, 1 mM DTT) at 4 °C overnight according to the manufacturer’s protocol. Subsequently, after confirming the completed removal of tags by SDS-PAGE, the resulting products were subjected to His-column purification to clear His-TEV protease and terminal TEV-H8-STREP tag peptide, which were also verified by SDS-PAGE stained by CBB R-250.

### 2.5. Bioassay of rTNFα Activity

The biological activity of the purified rh/rmTNFα proteins was assayed in culture murine L929 cells (RCB2619, supplied by Riken Cell Bank, Tsukuba, Japan) [30], which have been routinely employed for the cytotoxicity assay of TNFα. The cells were maintained in an RPMI1640 medium (Thermo Fisher Scientific, Waltham, MA, USA) supplemented with 10% fetal bovine serum (FBS, Sigma-Aldrich, St. Louis, MO, USA) at 37 °C with 5% CO_2_. Cells were seeded in 96 well-plates at a cell density of 2.5 × 10^4^ cells/well, and actinomycin D was added to each well at a concentration of 5 µg/mL. The *E. coli*-derived commercial (Peprotech, #315-01A, Cranbury, NJ, USA) or silkworm-derived rh/rmTNFα (with or without fusion tag removal) were added to each well with a gradient concentration. Viable cell density was measured at four days post-incubation using the Cell Counting Kit-8 (CCK8) assay to investigate and compare the cell cytotoxicity activity. All data were represented by the means ± standard error of three independent values.

## 3. Results and Discussion

### 3.1. Construction of rBmNPVs for the Production of rh/rmTNFα

Human and murine TNFα (hTNFα, mTNFα) precursor genes encode 233 and 235 amino acid residues, including the transmembrane region in 76 and 79 residues at N-terminus, respectively. The current study was designed to express the mature type of each TNFα, hTNFα (aa 77-233) and mTNFα (aa 80-235) [31,32]. Since both mature type TNFα cytokines execute biological functions as an extracellular form, we then employed a signal peptide from the silkworm 30K protein 6G (30K6G) to support the expressed protein to be secreted into silkworm hemolymph [23,27]. As for the location of terminal tags, since the N-terminal region is responsible for its binding to TNFR and purification tags at the N-terminus might have negative impacts on the protein function [33], a tandem PolyHistidine (8×His) and Strep-tag were attached to the C-terminus for facilitating the protein purification (Figure 1A). Both recombinant bacmid DNAs were constructed and further transfected into cultured silkworm BmN cells for generating recombinant baculoviruses to be used to inoculate a BmNPV-hypersensitive strain k45 [34,35].

### 3.2. Purification of rh/rmTNFα from Silkworm Serum

To evaluate the expression level of each TNFα protein in silkworm hemolymph, we performed the time course verification till the fifth day post-infection (dpi). Western blot results (Figure 1B) demonstrated that the expression (as arrowhead pointed) started detectable from 2 dpi in correct molecular weight positions (~21 kDa including C-terminal tags) and reached an expression peak at 4 dpi followed by a declining trend, suggesting 4–5 dpi is appropriate for sample collections.

**Figure 2:**
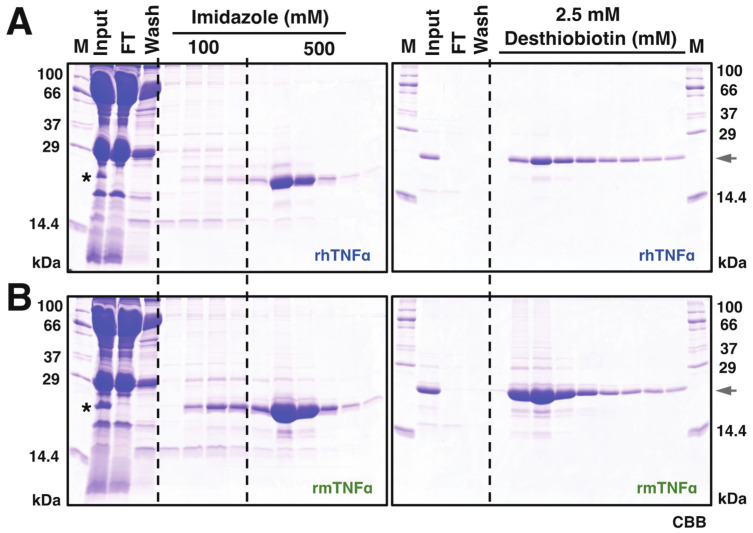
Purification of recombinant hTNFα (**A**) and mTNFα (**B**). Sera of rBmNPV-infection silkworm larvae were collected and treated as described in the Materials and Method section, which were further utilized for protein purification processes. A two-step purification procedure was performed via Nickel (His-Trap, (**A**,**B**), left panel) and Strep-Tactin ((**A**,**B**), right panel) affinity chromatography. M: molecular mass markers; FT: flow-through; Elution fractions from HisTrap purifications were further used in Strep-Tactin affinity chromatography. Protein samples were resolved on a 15% SDS-PAGE visualized by Coomassie Brilliant Blue (CBB) R-250. The asterisk indicates the visible band of expressed TNFα proteins from collected hemolymph samples. The arrowhead points out the expression of recombinant TNFα proteins.

Based on this observation, 10 mL serum from infected silkworms (~50 larvae) was harvested at 4 dpi for subsequent protein purifications. As shown in Figure 2, the secreted rhTNFα and rmTNFα proteins were visible on CBB-stained SDS-PAGE gel, indicating a considerable secretion level of both proteins in silkworm-BEVS. Subsequently, a two-step purification using HisTrap and StrepTrap was executed using the infected serum crudes as described in the “Materials and Methods” section. The SDS-PAGE verifications for rhTNFα (Figure 2A) and rmTNFα (Figure 2B) demonstrated that a significant protein yield was achieved in good purity, nearly 90%, as judged by image analysis of gels. Roughly, a total of ~6.55 mg rhTNFα (0.655 mg/mL sera, 0.131 mg/silkworm) and ~18.7 mg rmTNFα (1.87 mg/mL sera, 0.374 mg/silkworm) were confirmed after protein qualifications. It is noticed that the overall yield of rmTNFα is about 3-fold of rhTNFα, which could also be hinted from the Western blot results of Figure 1B using equal sera samples. The difference might be caused by secretion efficiency or protein stability.

It has been reported that recombinant human and murine TNFα has been successfully produced in other protein expression hosts with a milligram (mg) scale [8,10,12,13]. Compared with other protein expression systems such as the *E. coli* system, although most literature has claimed that up to mg protein per liter bacterial culture could be obtained, the expressed TNFα proteins formed inclusion bodies under which harsh denature reagents like 8 M urea before purification and protein refolding after purification are usually required to obtain active proteins [36,37]. More recently, several attempts using the *E. coli* expression system under optimized culture and induction conditions claimed that soluble human rhTNFα proteins could also be obtained in a large amount at 7.2 mg/L~1.26 g/L of culture medium [8,9]. In contrast, the silkworm-BEVS produces soluble TNFα proteins so that no refolding and other unnecessary processes are needed.

Previously, it has been reported that rhTNFα was able to be expressed in cultured Sf9 cells and *Plusia agnata* larvae as activity forms, indicating the usability of the baculovirus expression system for further production of TNFα proteins [11]. Basically, *B. mori* larvae are affordable and readily available in many Asian countries. The combination of silkworm and BEVS for producing POIs has been significantly improved in terms of productivity and quality since its initial development for the production of human alpha-interferon in silkworms [38]. The most attractive merits of silkworm-based BEVS are the low cost and flexibility for scale-up because each silkworm larva could be treated as an independent bioreactor [39]. In the current study, a yield of mg scale TNFα pure protein per mL sera after purification processes is a good starting point for future commercial purposes. What we found also suggests that silkworm-BEVS can be considered as a robust system to produce human and murine TNFα proteins.

### 3.3. Biological Activity

**Figure 3:**
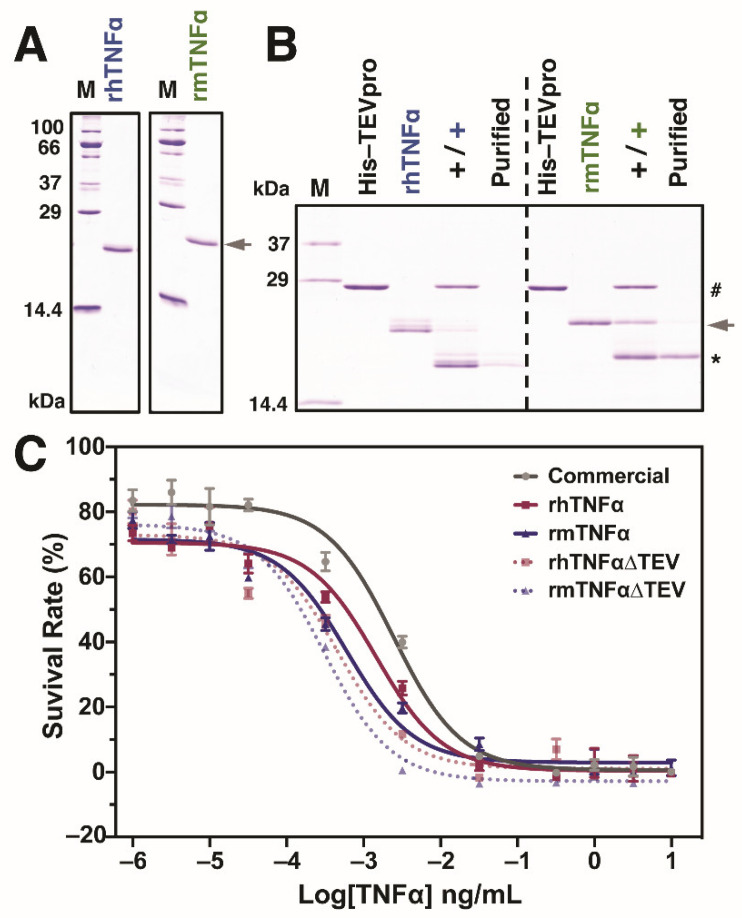
Biological activity of purified TNFα proteins using the cell viability assay in mouse fibroblast cells L929. The purified hTNFα and mTNFα proteins were shown with a decent purity in (**A**). To investigate whether or not the additional affinity tags negatively impact the protein activity, both proteins were digested with TEV proteinase for activity verification (**B**). After incubation, the resulting products were further purified via binding to His-beads for removing TEV protease and undigested TNFα contaminations. # indicates His-tagged TEV proteinase (His-TEVpro); the arrowhead indicates hTNFα and mTNFα with affinity tags; * indicates fully digested TNFα without C-terminal affinity tags. (**C**) Cell Counting Kit-8 (CCK8) assay was performed to investigate and compare commercial *E. coli*-derived TNFα (murine type, grey line) with silkworm-derived TNFα proteins with (hTNFα and mTNFα, red and blue line) or without affinity tags (hTNFαΔTEV and mTNFαΔTEV, red and blue dotted line). All data were represented by the mean ± standard error of four independent values.

One of the most attractive activities of TNFα proteins is cytotoxicity against various cancer cells in vitro and in vivo [4,40,41]. Subsequently, we focused on the evaluation of the biological activity of produced rTNFα proteins in the murine L929 fibroblast cells. To avoid the potential effects from storage buffer conditions during protein purifications, all the purified proteins were dialyzed against the PBS buffer and verified by SDS-PAGE before cell assays, as confirmed in Figure 3A. In the current study, we also investigated the influence of C-terminal tags on the TNFα functions [33,42]. As plotted in Figure 3B, terminal tags from both TNFα proteins were successfully removed (termed TNFα∆TEV) after incubation with TEV proteinase. All PBS-buffered protein samples, including a positive control from commercial *E. coli*-derived murine TNFα, were then assayed together with the L929 cells in a 10^-6^~10 ng/mL concentration range. The results from Figure 3C demonstrated that all of the median effective dose (ED_50_) after curve fitting: 2.46 × 10^−3^ ng/mL (commercial mTNFα), 1.45 × 10^−3^ ng/mL (rhTNFα), 0.60 × 10^−3^ ng/mL (rhTNFα∆TEV), 0.46 × 10^−3^ ng/mL (rmTNFα), 0.31 × 10^−3^ ng/mL (rmTNFα∆TEV), respectively. Interestingly, we observed that the silkworm-derived murine rmTNFα/rmTNFα∆TEV showed better performance over the *E. coli*-derived product since a lower ED_50_ value was obtained in murine L929 cells. However, more cancerous and noncancerous cells should be investigated to see whether there is a cell type-depended activity of purified TNFα proteins. Generally, the activity of human TNFα is lower than murine TNFα when comparing the ED_50_ of rhTNFα and rmTNFα from silkworm-BEVS, indicating that TNFα might hold certain species-specificity on different cells.

Moreover, it is interesting to find that the ED_50_ decreased after removing the C-terminal tags in both TNFα proteins, suggesting the attached tags might negatively influence the protein activities [33,42]. It is believed that terminal tags are beneficial for protein detections and purifications. However, it is also troublesome when the extra tags affect the protein conformation and stability, attenuating the protein function in the end [43]. To date, most of the recombinant TNFα proteins from various systems have been purified with affinity tags such as Polyhistidine and Glutathione S-transferase (GST) [8,14,44,45]. Based on our results, it might be better to remove the terminal tags to achieve the best performance of the cytotoxicity of TNFα. On the other hand, other efforts such as the purification design and technology for non-tagged TNFα protein from silkworm protein crude are also preferred [42,46]. Taken together, we successfully produced both active human and murine TNFα proteins in silkworm-BEVS, which could meet the requirements for further pharmaceutical and clinical trials.

## Data Availability

Data is contained within the article.
